# Myotomy for myocardial bridge utilizing harmonic scalpel

**DOI:** 10.1016/j.ijscr.2023.108783

**Published:** 2023-09-06

**Authors:** Bryan A. Miles, Joseph A. Lahorra

**Affiliations:** Department of Cardiothoracic Surgery, Cleveland Clinic Akron General, Akron, USA

**Keywords:** Case report, Myocardial bridge, Myotomy, Harmonic scalpel

## Abstract

**Introduction:**

Myocardial bridge is defined as epicardial coronary arteries that course through the myocardium. While frequently asymptomatic, it can present on a spectrum from stable to life threatening angina. Medical management is often successful, but failure requires stenting or bypass, both of which are inferior to myotomy in appropriate surgical candidates, the former due to morbidity and the later theoretically due to competitive flow.

**Presentation of case:**

We present an otherwise healthy 50 year old gentleman with myocardial bridge refractory to medical management who was effectively managed via myotomy performed with the harmonic scalpel, enjoying complete relief of previous exertional chest pain.

**Discussion:**

Historically, myotomy has been described sharply and with electrocautery. Compared to the harmonic scalpel, these techniques risk poor hemostasis and damage to the underlying left anterior descending artery, not to mention their inefficiency in terms of operative speed.

**Conclusion:**

In appropriately diagnosed patients, who are also suitable surgical candidates, myotomy, specifically with the harmonic scalpel, has short-term, intra-operative benefits of better hemostasis, protection of underlying left anterior descending artery and heart cavity, and improved operative efficiency. Given the lack of long-term symptomatic data on different myotomy techniques it is difficult to make comparisons of this nature.

## Highlights

• 50 year old man with exertional chest pain secondary to left anterior descending myocardial bridge
• Failed medical management
• Underwent myotomy using Harmonic Scalpel versus traditional sharp or electrocautery methods
• Enjoyed resolution of symptoms

## 1 Introduction

Myocardial bridge (MB) is defined as epicardial coronary arteries that course through the myocardium. A somewhat rare finding that is infrequently symptomatic, it can present on a spectrum from stable angina to sudden cardiac death secondary to arrhythmia [[4], [5], [6]]. Medical management is often successful, however when that fails, intervention may be indicated [7,8]. Stenting or bypass are generally not as successful as surgical myotomy [[9], [10], [11], [12], [13], [14], [15], [16], [17], [18], [19], [20], [21], [22], [23], [24], [25]]. We report our experience using the ultrasonic scalpel (Harmonic Scalpel, Ethicon End of Surgery, Raritan, New Jersey) as a safe, surgically efficient, and effective surgical technique to establish myotomy of MB.

## 2 Presentation of case

**Fig. 1:**
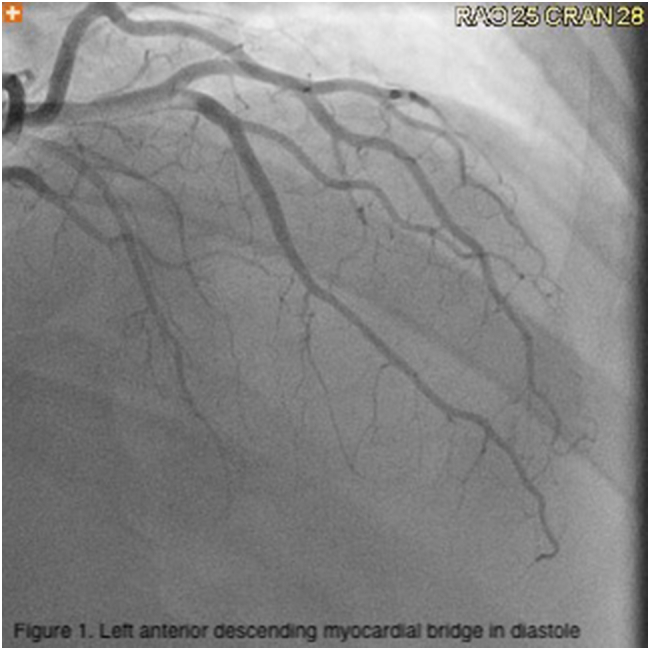
Left anterior descending myocardial bridge in diastole.

**Fig. 2:**
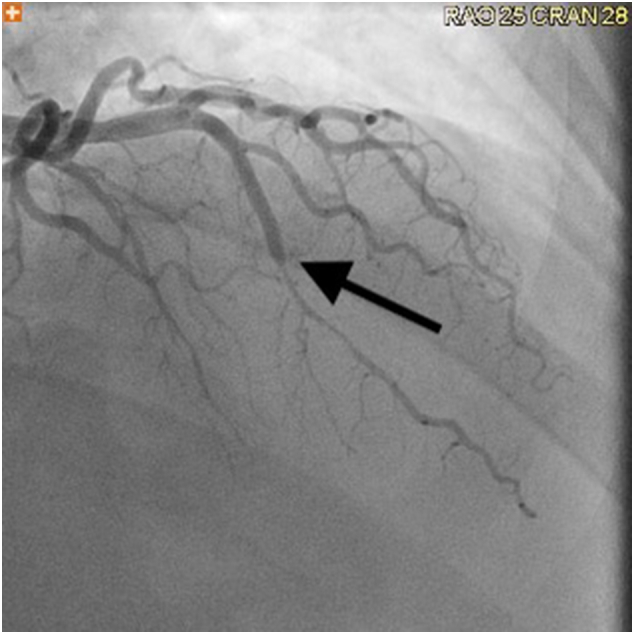
Left anterior descending myocardial bridge in systole.

**Fig. 3:**
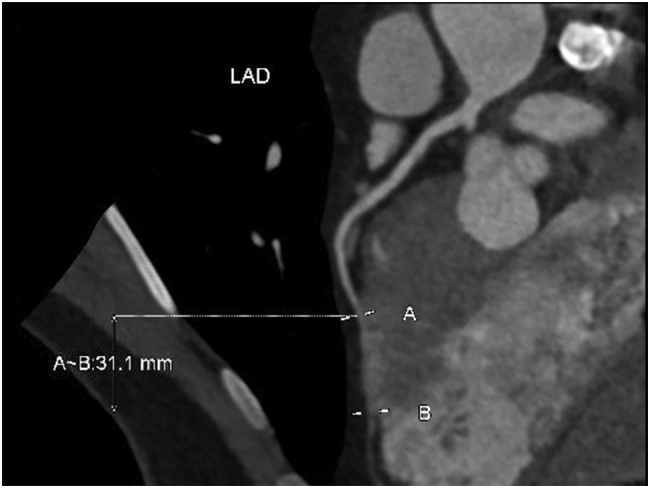
CTA showing 3.1 mm LAD myocardial bridge.

A 50 year old man with a history of hypertension presented to the emergency department with recurrent, debilitating exertional chest pain. Electrocardiogram and troponins were unremarkable (Fig. 1, Fig. 2). Nuclear medicine stress test was also negative for ischemia. Echocardiogram showed normal function without valvular abnormalities. Computed tomography angiography (CTA) revealed 3.1 cm long segment of left anterior descending (LAD) intramyocardial bridging with no blood flow during systole, but distal reconstitution during diastole (Fig. 3). Cardiac catheterization similarly showed myocardial bridge of LAD causing significant flow restriction during systole. Symptoms, which significantly interfered with the patient's profession as a firefighter, as well as simple activities of daily living, persisted despite medical management with metoprolol and amlodipine. Surgical myotomy was then recommended due to failure of medical management and severity of disease, despite no ischemia, seen on diagnostic modalities, including CTA and cardiac catheterization. These are included below.

Following routine sternotomy, cardiopulmonary bypass (CPB) was initiated via the aorta and right atrium. Myocardial protection was provided with antegrade cardioplegia. The LAD and its intramyocardial segment were identified. Using a fine mosquito, the distal aspect of the bridge was elevated off the underlying LAD as it emerged. Once a plane was initiated, the non-active tine of the harmonic scalpel was inserted between the LAD posteriorly and the MB anteriorly. Division of the MB was accomplished with multiple applications of the harmonic scalpel, proceeding in a distal to proximal fashion. The harmonic scalpel divided the bridge, while simultaneously providing hemostasis and protection to the underlying LAD. Following completion of the myotomy, the patient was successfully weaned off cardiopulmonary bypass. The remainder of his hospital course was uneventful with discharge on post operative day four. Ultimately, the patient enjoyed complete relief of previously described severe symptoms.

## 3 Discussion

We highlight the case of an otherwise healthy 50-year-old man with MB refractory to medical management, who was effectively managed via myotomy performed with the harmonic scalpel. Pathologic studies have found a mean frequency of MB of 25 %, while angiographic studies have reported the prevalence as 1.7 %, almost always being confined to the LAD [1]. In a study of 100 patients using computed tomography angiography (CTA), MB of coronary arteries was found in 34 %, but only approximately one-third of these showed systolic compression [2]. Although coronary artery flow occurs in diastole, quantitative coronary angiography combined with intravascular ultrasound (IVUS) and Doppler flow measurements have shown delayed relaxation of the MB segment in diastole [3]. Patients with MB with significant compression may present with silent ischemia, stable angina, acute coronary syndromes, stress cardiomyopathy, or malignant arrhythmias, possibly leading to sudden cardiac death [[4], [5], [6]]. During diagnostic coronary angiography, MB is recognized as compression of a segment of coronary artery during systole, but some degree of reversal during diastole. This dynamic nature differentiates it from fixed coronary stenosis. Only symptomatic patients require treatment, with the vast majority responding well to pharmacologic therapy, specifically beta blockers, iavbradine, and possibly nondihydropyridine calcium channel blockers [7,8]. Only those refractory to medical therapy should be considered for percutaneous or surgical treatment. Intracoronary stent placement provides the lumen of bridged segments stability against external compression and ultimately improves coronary hemodynamics. However, recurrent anginal symptoms due to in-stent restenosis, in-stent thrombosis, perforation, and stent fracture are reported, thus stent placement for medically refractory patients should be utilized only in poor surgical candidates [[9], [10], [11], [12], [13], [14], [15]]. Surgical intervention has consisted of myotomy, coronary artery bypass grafts, or a combination of the two [[15], [16], [17], [18], [19], [20], [21], [22], [23], [24], [25]]. Coronary bypass grafting has been described, but with inferior results due to competitive flow and subsequent graft failure. The classic description of myotomy is division of the muscle fibers with sharp dissection or electrocautery [26]. Compared to harmonic scalpel, these are both less operatively efficient and hemostatic, but more importantly, less safe as they risk damage to the underlying LAD or even entry into the heart cavity itself. The harmonic scalpel employs ultrasonic energy, which is ultimately converted to mechanical energy at the active tine. This results in a high-grade frictional force via the active tine, which simultaneously cauterizes and divides the tissue. Ultimately, this precise dissection highlights the safe, efficient, and effective attributes of the ultrasonic scalpel during this procedure [27]. While our approach involved CPB, this is not an absolute with the harmonic scalpel. However, we choose this though since it would allow for optimization of the stated benefits of the harmonic scalpel, namely precision. Our work has been reported in line with the SCARE criteria [28].

## 4 Conclusion

In appropriately diagnosed patients, who are also suitable surgical candidates, myotomy, specifically with the harmonic scalpel, provides short-term, intra-operative benefits of better hemostasis, protection of underlying LAD and heart cavity, and improved efficiency, with respect to operative time. We believe this approach is highly replicable and can benefit similar patients worldwide.

## Funding

This research did not receive any specific grant from funding agencies in the public, commercial, or not-for-profit sectors.

## Consent

Cleveland Clinic Akron General Institutional Review Board approved the waiver for the need of ethics approval for this study.

## Declaration of competing interest

Accordingly, there are no conflicts of interest.
